# Temperature Dependent Excitonic Transition Energy and Enhanced Electron-Phonon Coupling in Layered Ternary SnS_2-x_Se_x_ Semiconductors with Fully Tunable Stoichiometry

**DOI:** 10.3390/molecules26082184

**Published:** 2021-04-10

**Authors:** Der-Yuh Lin, Hung-Pin Hsu, Chi-Feng Tsai, Cheng-Wen Wang, Yu-Tai Shih

**Affiliations:** 1 Department of Electronic Engineering, National Changhua University of Education, Changhua City 500, Taiwan; dylin@cc.ncue.edu.tw (D.-Y.L.); M0553007@gm.ncue.edu.tw (C.-F.T.); M0853001@gm.ncue.edu.tw (C.-W.W.); 2 Department of Electronic Engineering, Ming Chi University of Technology, Taishan, New Taipei City 243, Taiwan; 3 Department of Physics, National Changhua University of Education, Changhua City 500, Taiwan; ytshih@cc.ncue.edu.tw

**Keywords:** 2D semiconductors, chemical–vapor transport, van der Waals

## Abstract

In this study, a series of SnS_2-x_Se_x_ (0 ≤ x ≤ 2) layered semiconductors were grown by the chemical–vapor transport method. The crystal structural and material phase of SnS_2-x_Se_x_ layered van der Waals crystals was characterized by X-ray diffraction measurements and Raman spectroscopy. The temperature dependence of the spectral features in the vicinity of the direct band edge excitonic transitions of the layered SnS_2-x_Se_x_ compounds was measured in the temperature range of 20–300 K using the piezoreflectance (PzR) technique. The near band-edge excitonic transition energies of SnS_2-x_Se_x_ were determined from a detailed line-shape fit of the PzR spectra. The PzR characterization has shown that the excitonic transitions were continuously tunable with the ratio of S and Se. The parameters that describe the temperature variation of the energies of the excitonic transitions are evaluated and discussed.

## 1. Introduction

Two-dimensional (2D) layered materials have attracted tremendous attention owing to their crucial role in the development of next-generation electronic devices. 2D layered structures comprise covalently bonded atomic sheets that are vertically held together by the van der Walls forces. The successful exfoliation of each atomically thin layer of such 2D materials and the layer-by-layer heterogeneous assembly enable us to realize a wide range of electronic and optoelectronic applications down to the atomically thin layer scale. Thus far, 2D materials such as graphene [1], black phosphorus [2], boron nitride [3], silicone [4], and transition metal dichalcogenides (TMDs) [5,6,7] are considered to be the possible materials for the future electronic circuit applications due to their unique electrical and optical properties. Among these, TMDs are believed to be one of the promising candidates for the future electronics because of their semiconducting behavior. To date, the TMDs exhibiting MX_2_ (M = Mo or W; X = S or Se) type configurations, are the most investigated due to their interesting physical and optical properties for the 2D based technology [8,9,10,11]. The few-layer field effect transistor [12], phototransistor [13], p-n junction diode [14], solar cells [15], radio frequency electronics [16], and fin-shaped field effect transistor [17] were successfully fabricated using TMDs, demonstrating their potential for the next generation electronic devices. Future application such as the implementation of 1-bit microprocessor based on TMDs was also realized [18]. The successful demonstration of potential applications of the above mentioned MX_2_ type TMDs in 2D electronics intrigued interest in other prospective TMDs. However, the study of other TMDs are still required for the 2D based device technology. Recently, the Group-IVA TMDs, like SnS_2_ and SnSe_2_, were proposed for the 2D electronic device applications. The unique layered structures of SnS_2_ and SnSe_2_ make them potential candidates in the 2D nanoelectronics and optoelectronics. The potential application of few-layered SnS_2_ as transistors [19] and photodetectors [20] was reported. Further studies with high mobility [21] and on/off ratios [22] of the SnSe_2_ transistors were also reported. However, the band gap flexibility of these binary alloys is limited. The ternary alloys of the compounds have the tunable band gap that is critical for optical device applications. Thus far, only the basic properties of the ternary SnS_2-x_Se_x_ were reported [23,24]. Further investigations of SnS_2-x_Se_x_ as flexible sensors [25] and phototransistors [26] were also reported. However, in spite of their potential applications, the temperature dependent band-edge excitonic transition properties are still obscure. Hence, further studies on the SnS_2-x_Se_x_ alloys focusing on the temperature dependence of energies of the band-edge excitons are not only interesting, but also necessary for the 2D devices design consideration.

In this study, we report the investigation of the structural properties of SnS_2-x_Se_x_ alloys by X-ray diffraction (XRD), Raman spectroscopy, and electron probe X-ray micro-analyzer (EPMA) characterization techniques. A detailed study of the temperature dependence of the piezoreflectance (PzR) measurements of SnS_2-x_Se_x_ single crystals in the temperature range 20–300 K was also investigated. The PzR spectra are fitted with the Aspnes equation of the first-derivative Lorentzian line shape [27] to determine temperature dependence of direct band edge excitonic transitions of SnS_2-x_Se_x_ crystals. The parameters that describe the temperature variation of the energies of the excitonic transitions are evaluated and discussed.

## 2. Experimental

The SnS_2-x_Se_x_ layered single crystals were grown by chemical–vapor transport method. The chemical transport was achieved with I_2_ (5 mg/cm^3^) as transport agent, and the constituent elements consisting of high purity (Sn:99.99%, S:99.99%, and Se:99.99%) were sealed in a quartz ampoule under high vacuum. The elements Sn, S, and Se were weighed to fit the molar ratio of SnS_2-x_Se_x_ (0 ≤ x ≤ 2), and the quartz ampoule pressure was maintained at approximately 2 × 10^−5^ torr during the sealing process. The quartz ampoule (30 mm OD × 25 mm ID × 28 cm) was then introduced into a two-zone furnace and heat to 800 °C in the high temperature zone and 650 °C in the low temperature zone for a duration of 14 days. The crystals exhibited the shape of 20–100 µm thin layered plates with a surface area up to 5~10 mm^2^. In this work, six SnS_2-x_Se_x_ crystals were grown with a nominal composition x varied as 0, 0.4, 0.8, 1.2, 1.6, 2. The lattice structure and alloy compositions for all the SnS_2-x_Se_x_ crystals were confirmed by XRD and EPMA, respectively. The X-ray diffraction (XRD) studies were carried out using a Shimadzu XRD6000 X-ray diffractometer (Shimadzu, Kyoto, Japan) using CuKα (λ = 1.5406 Å) radiation with independent dual axis θ–2θ linkage drive. The Raman spectroscopy was carried out on 3D Nanometer Scale Raman spectrometer (Tokyo Instruments, Nanofinder 30) with 488 nm laser. The laser power was operated at ~1 mW to avoid the laser heating effects. High resolution transmission electron microscopy (HRTEM) images and selected area electron diffraction (SAED) patterns were recorded to characterize the lattice structure of SnS_2-x_Se_x_ crystals by PHILIPS CM-200 TWIN FE-TEM.

The PzR measurements were achieved by gluing the thin single crystal specimen on a 0.15 cm thick lead-zirconate-titanate (PZT) piezoelectric transducer driven by a 400 Vrms sinusoidal wave at ~200 Hz. The alternating expansion and contraction of the transducer subjected the sample to an alternating strain with a typical rms Δl/l value of ~10^−5^. A 200 W tungsten-halogen lamp filtered by a PTI model 102 0.25 m monochromator provided the monochromatic light. The reflected light was detected by an EG&G HUV-2000B silicon detector. The direct-current output of the silicon photodiode was maintained constant by a servo mechanism of a variable neutral density filter. A SIGNAL RECOVERY model 7265 dual-phase lock-in amplifier was used to measure the detected signal. A close-cycle cryogenic refrigerator equipped with a digital thermometer controller was used to control the measurement temperature between 20 and 300 K with a temperature stability of 0.5 K or better.

## 3. Results and Discussion

**Figure 1:**
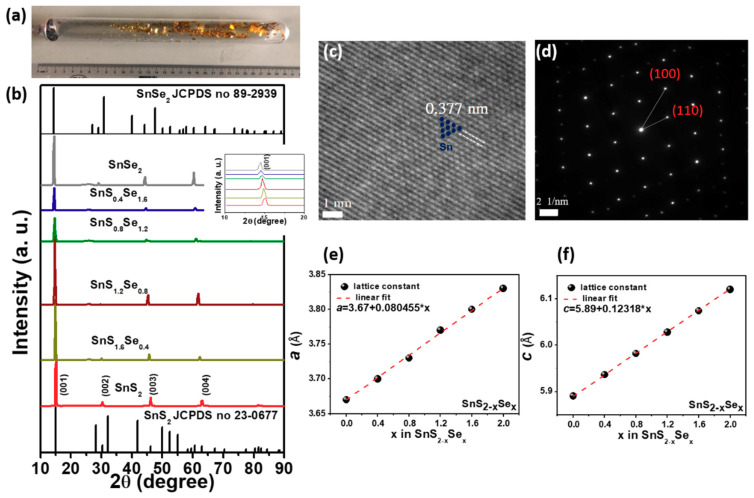
(**a**) Macroscopic single crystals synthesized from the CVT method. (**b**) XRD patterns of SnS_2-x_Se_x_ layered crystals with varying Se composition. The inset is the magnified (001) peak. (**c**) High resolution transmission electron microscopy (HRTEM) image and (**d**) selected area electron diffraction (SAED) pattern. (**e**,**f**) Variation of lattice constants *a* and *c* of SnS_0.8_Se_1.2_ crystal.

Figure 1a showed the macroscopic single crystals synthesized from the chemical–vapour transport (CVT) method. Figure 1b depicts the XRD patterns of SnS_2-x_Se_x_ layered single crystals as a function of Se composition. The major diffraction peaks of SnS_2-x_Se_x_ crystals are labelled for hexagonal unit cell of the CdI_2_ type that belongs to the hexagonal Pbnm space group (JCPDS No.23-0667 for SnS_2_ and No. 89-2939 for SnSe_2_). The strong diffraction peak at ~15° is assigned to the (001) plane, other small diffraction peaks are assigned to the (002), (003), (004), and (005) planes [28]. The main peak positions of SnS_2-x_Se_x_ crystals gradually shifted to lower angles with increasing Se composition (inset Figure 1a). It is noted here that only the (001) planes were observed in the presented study. This observation put in evidence a preferential orientation with (00l) planes parallel to the surface in layered SnS_2-x_Se_x_ alloys. We also characterized the crystal structure by HRTEM for all SnS_2-x_Se_x_ crystals. Figure 1c,d shows the representative HRTEM image and SAED patterns of SnS_0.8_Se_1.2_ crystal. It is known that the Z-contrast of Sn (Z = 50) is heavier than Se (Z = 34) and S (Z = 16), which is why the brightness of Sn sites in HRTEM image is larger than that of Se and S sites. The Sn sites are marked with light blue points on the HRTEM image for clear recognition. The lattice constants *a* and *c* of SnS_2-x_Se_x_ crystals deduced from the HRTEM and XRD, respectively, are listed in Table 1 along with the nominal and real composition of SnS_2-x_Se_x_ measured by EPMA. The results indicated that the real composition of the grown crystals are consistent with the nominal stoichiometry. Figure 1e,f show that lattice constants (*a* and *c*) increase linearly with increasing Se content. Such linear evolution of lattice constants with composition were observed, which follows the Vegard’s law.

**Figure 2:**
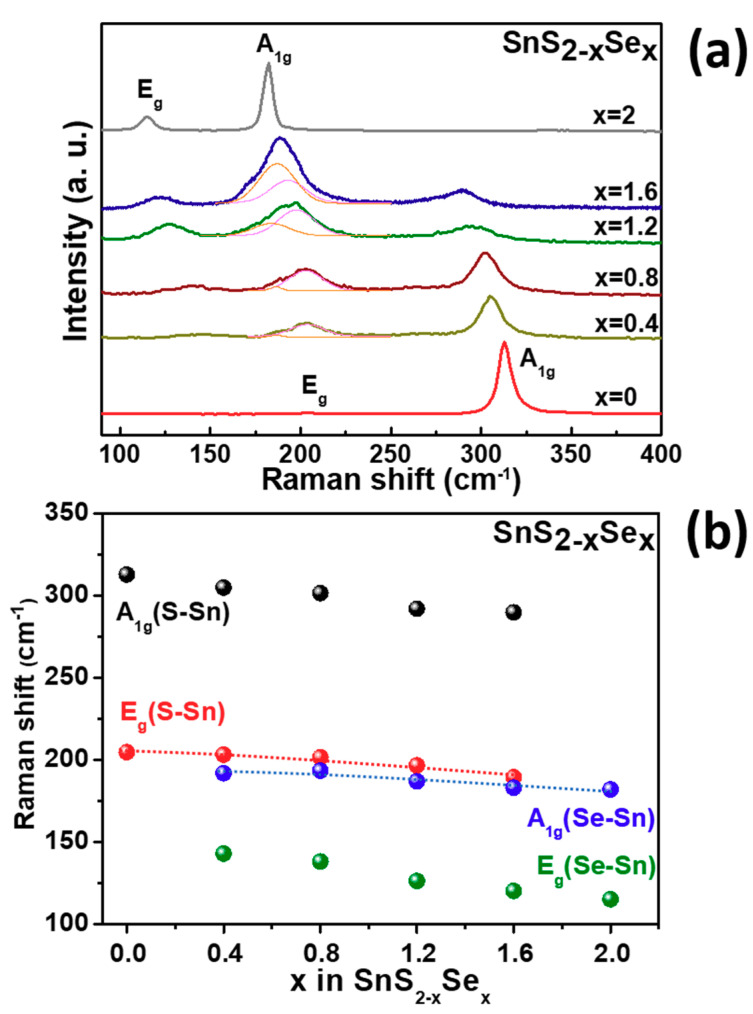
(**a**) Composition-dependent Raman spectra of SnS_2-x_Se_x_ layered crystals. The pink and orange curves correspond to the adjustments of the E_g_(S-Sn) and A_g_(Se-Sn) peaks, respectively. (**b**) Evolution of Raman peak positions of SnS_2-x_Se_x_ in the range of 100–400 cm^−1^. The dashed lines are guided by eyes.

Figure 2a shows the Raman spectra of all the synthesized SnS_2-x_Se_x_ single crystals. The observed composition dependent vibration modes of SnS_2-x_Se_x_ were assigned as A_1g_ (Se-Sn), E_g_ (Se-Sn), A_1g_ (S-Sn), and E_g_ (S-Sn). The intensity of S-Sn related Raman modes decrease with increasing Se composition. Meanwhile the vibration modes of Se-Sn enhance gradually. In Figure 2b, the statistical analysis of Raman vibration modes of the SnS_2-x_Se_x_ single crystals are depicted. The Raman frequencies of A_1g_ and E_g_ vibration modes showed a red-shift trend with increasing Se composition [29]. The evolution of the Raman frequency shifts and intensity changes with the S/Se atomic ratio were consistent with those of MoS_2-x_Se_x_ [30], WS_2-x_Se_x_ [31], and HfS_2-x_Se_x_ [32] alloys. The XRD measurements and Raman spectra provided the signature for identifying the crystal structure and material phase of SnS_2-x_Se_x_ layered semiconductors.

**Figure 3:**
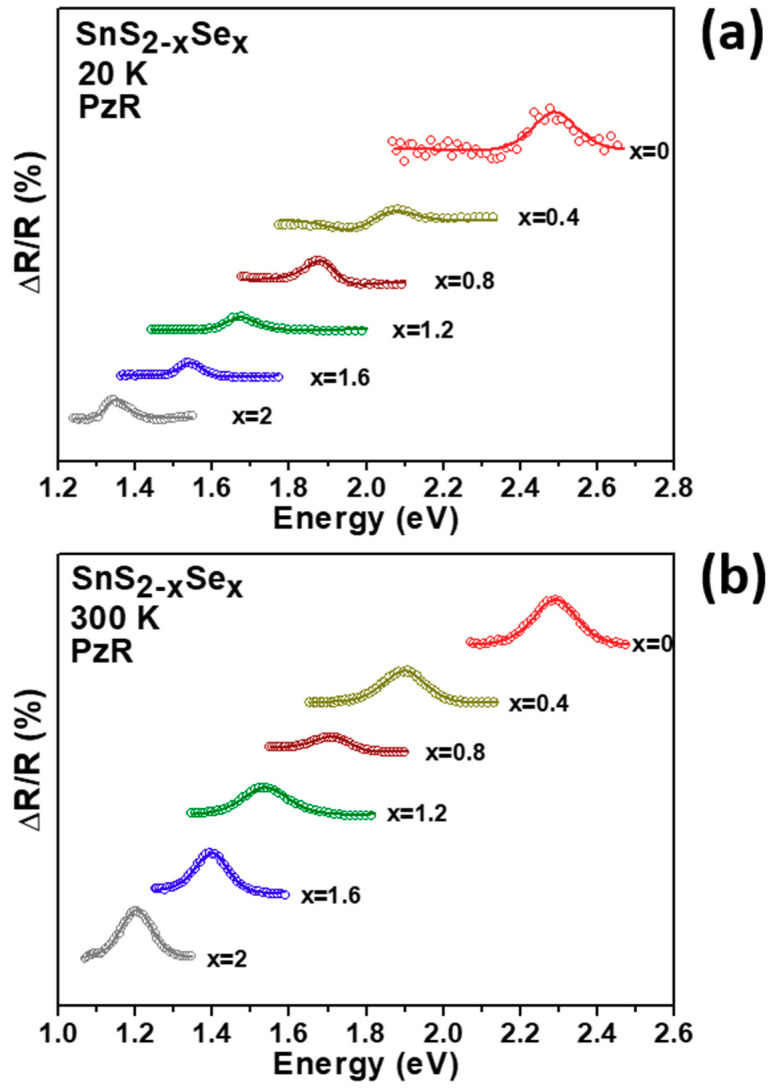
The experimental PzR spectra (dashed curves) of SnS_2-x_Se_x_ layered crystals at (**a**) 20 K and (**b**) 300 K. The solid curves are least-squares fits to Equation (1) which yields the excitonic transition energies.

Figure 3a,b shows the PzR spectra in the vicinity of the direct band edge excitonic transitions of SnS_2-x_Se_x_ crystals at 20 and 300 K, respectively. The experimental data in dotted lines reveal the oscillator of PzR spectra in SnS_2-x_Se_x_ crystals. In order to determine the direct band edge excitonic transitions from PzR spectra, we have fitted the experimental data with the first derivative Lorentzian line-shape (FDLL) function. The solids lines are the east-squares fits to the FDLL function of the form [27]:

(1) ΔRR=Re[∑i=1nAexejφex(E−Eex+jΓex)−2]

where $A_{i}^{ex}$ and $\phi_{i}^{ex}$ are the amplitude and phase of the line shape, $E_{i}^{ex}$ and $\Gamma_{i}^{ex}$ are the energy and broadening parameters of the excitonic transitions, respectively. The estimated excitonic transition energies ($E_{i}^{ex}$) of SnS_2-x_Se_x_ alloys were ranging from 1.207 eV for SnSe_2_ to 1.406, 1.535, 1.713, 1.899, and 2.289 eV with increasing S content at 300 K. From the PzR spectra of SnS_2-x_Se_x_ crystals, the excitonic transition energies vary smoothly with the Se composition. From the PzR spectra of SnS_2-x_Se_x_ crystals, we can observe that the excitonic transition energies vary smoothly with the Se composition. This result indicates that the fully tunable chemical compositions modification of ternary SnS_2-x_Se_x_ was achieved.

**Figure 4:**
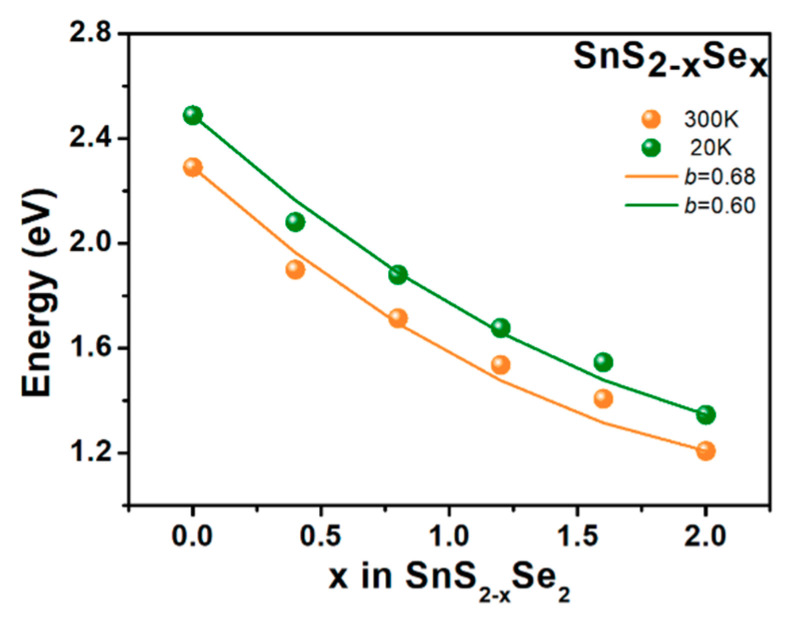
Composition dependent excitonic transition energies for SnS_2-x_Se_x_ layered crystals at 300 K. The solid curves are least-squares fits to Equation (2).

Figure 4 depicts the sulfur composition dependence of the excitonic transition energies at 300 K. The composition-dependence of excitonic transition energies of SnS_2-x_Se_x_ crystals vary smoothly with x. Hence, the dependence of excitonic transition energies for SnS_2-x_Se_x_ crystals can be described by the following conventional bowing equation [33]:

(2) $$E^{ex}\left(x\right)=\frac{x}{2}E^{ex}\left({\mathrm{SnSe}}_{2}\right)+\left(1-\frac{x}{2}\right)E^{ex}\left({\mathrm{SnS}}_{2}\right)-b\frac{x}{2}\left(1-\frac{x}{2}\right)$$

where, $E_{}^{ex}$ is excitonic transition energy, *b* is the bowing constant. The solid line represents the fitted data for the composition-dependence of excitonic transition energy of the ternary semiconductor alloys according to the generalized equation. In this study, *b* was determined to be 0.60 and 0.68 eV for temperature at 20 and 300 K, respectively, which is in a reasonable agreement with the previous published values in the range of 0.03 to 1.10 eV [28,34,35].

**Figure 5:**
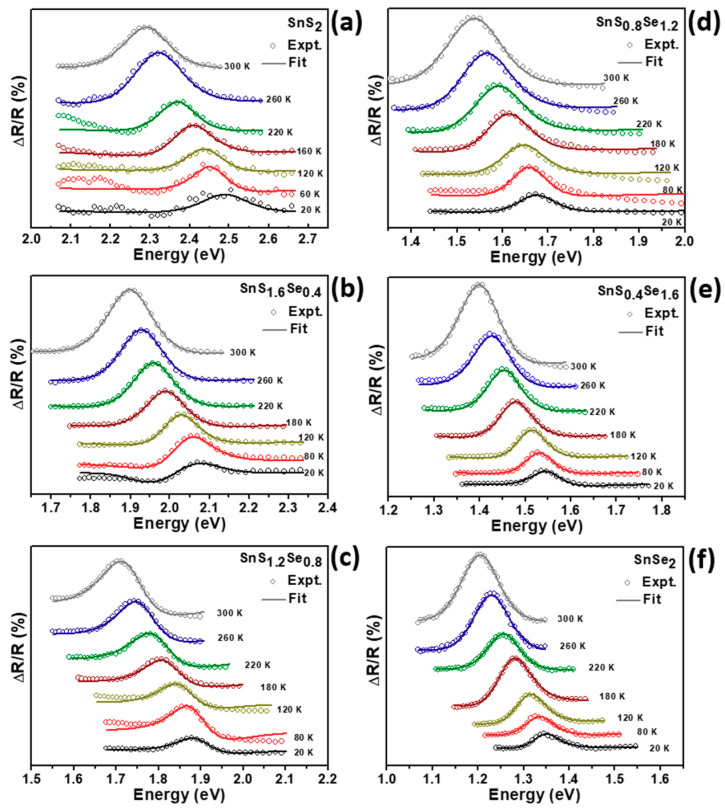
(**a**–**f**) Experimental PzR spectra at various temperatures of the SnS_2-x_Se_x_ layered crystals for S content *x* ranging from 0 to 2. The solid curves are the least-squares fits to Equation (1).

Figure 5 show the experimental temperature dependent PzR spectra of SnS_2-x_Se_x_ crystals at various temperatures between 20 and 300 K. The dotted lines are the experimental curves and the solid lines are the fitted spectral data to Equation (1) with *n* = 2, that yields the excitonic transition energies. As a general property of most semiconductors, when the measuring temperature is increased, the excitonic transitions in the PzR spectra exhibit an energy red-shift characteristic due to the band gap thermal shrinkage. In order to study the behavior of the temperature dependence of various excitonic transitions, we further studied the temperature dependence properties in the range of 20–300 K by O’Donnell and Chen and Bose–Einstein statistical model.

**Figure 6:**
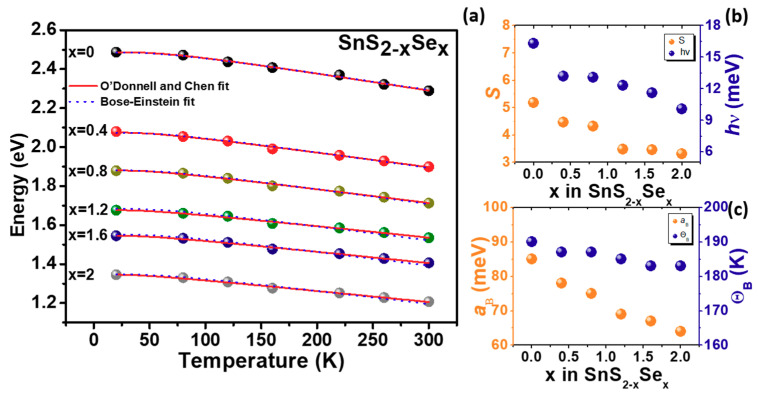
(**a**) The temperature dependence of the excitonic transition energies of SnS_2-x_Se_x_ layered crystals. The solid curves are least-squares fits to Equation (3) and the dashed curves are least-squares fits to Equation (4). (**b**,**c**) The parameters obtained from Equations (3) and (4) that describe the temperature variation of SnS_2-x_Se_x_ layered crystals were also plotted.

Figure 6 shows the temperature dependence of *E^ex^*(*T*) extracted from PzR of SnS_2-x_Se_x_ crystals. The solid lines are the least-squares fits to the O’Donnell and Chen semi-empirical relationship [36]:

(3) $$E^{ex}(T)=E^{ex}(0)-S(hv)[\coth(<hv>/2k_{B}T)-1]$$

where, *E^ex^*(0) is the excitonic transition energies at 0 K. The constant *hv* is the average phonon energy, and S is the dimensionless electron-phonon coupling constant. The obtained values of *E^ex^*(0), *hv*, and S for SnS_2-x_Se_x_ crystals are listed in Table 2.

We have also fitted the experimental data to a Bose–Einstein expression (dashed lines) [37]:

(4) $$E^{ex}(T)=E^{ex}(0)-\frac{2a_{B}}{\left[\exp(\Theta_{B}/T)-1\right]}$$

where, *E^ex^*(0) are the excitonic transition energies at 0 K, *a_B_* represents the strength of the electron (exciton)-average phonon interaction, and $\Theta_{B}$ corresponds to the average phonon temperature. The fitted values obtained for the various parameters are also presented in Table 2, together with the parameters for the transition energies of SnS_2_, SnSe_2_, SnS_1.4_Se_0.6_ [34,38], and SnS [39] measured by absorption and photoreflection techniques for comparison.

The result of the fit to the O’Donnell and Chen and Bose–Einstein statistical model can be compared in the high temperature limit where they reduce to:

(5) $$E^{ex}(T)=E^{ex}(0)-2Sk_{B}T$$

(6) $$E^{ex}(T)=E^{ex}(0)-2a_{B}T/\Theta_{B}$$

The above equations yields, *S* = $a_{B}/k_{B}\Theta_{B}$. By substituting the values of *a_B_* and $\Theta_{B}$ obtained from the Bose–Einstein fit. We calculated *S* = 5.19 which agrees well with the value *S* = 5.18 obtained from O’Donnell and Chen statistical model for SnS_2_. Comparison of the numbers presented in Table 2 show that this relation is satisfied approximately for SnS_2-x_Se_x_ crystals. The results indicate the consistency between the parameters obtained by O’Donnell and Chen and Bose–Einstein statistical model. From Equation (5), it follows that the excitonic transition energies variation with temperature [*dE^ex^/dT*] is proportional to −2*Sk_B_* at high temperatures. The calculated value of −2*Sk_B_* for the excitonic transition energies of SnS_2-x_Se_x_ crystals equals to 0.89, −0.77, −0.74, −0.60, −0.59, and −0.57 meV/K for *S* content x ranging from 0 to 2, that is in a reasonable agreement with the value of [*dE^ex^/dT*] = 0.82, −0.73, −0.70, −0.61, −0.58, and −0.56 as obtained from the linear extrapolation of the high temperature (120~300 K) PzR experimental data. The temperature dependence parameters indicate the enhanced electron–phonon coupling with increasing the sulfur composition.

## 4. Conclusions

In conclusion, a series of TMD SnS_2-x_Se_x_ layered crystals were successfully grown by the chemical vapor transport method. Both crystal structure and material phase of SnS_2-x_Se_x_ layered single crystals were characterized by XRD spectra and Raman spectra. The temperature dependence of the excitonic transition energies of SnS_2-x_Se_x_ layered single crystals were characterized by PzR technique in the temperature range between 20 and 300 K. The tunable excitonic transition energies of SnS_2-x_Se_x_ was achieved by varying the ratio of S and Se. The Se contents dependent excitonic transition energies could be described by an expression including bowing parameters. The temperature dependence of SnS_2-x_Se_x_ excitonic transition energies was measured and the data were fit to the semi-empirical O’Donnell and Chen and Bose–Einstein models. The parameters that describe the temperature dependence of the excitonic transition energies indicated an enhanced electron–phonon coupling with the increasing sulfur composition.

## Figures and Tables

**Table 1:** The composition x of the SnS_2-x_Se_x_ alloys assessed by electron probe X-ray micro-analyzer (EPMA) and lattice constants *a* and *c* deduced from HRTEM and XRD, respectively.

	Measured Composition (%)			Lattice Constant (Å)	
SnS_2-x_Se_x_	Sn	S	Se	*a*	*c*
SnS_2_	33.46	66.54		3.67 ± 0.02	5.89 ± 0.02
SnS_1.6_Se_0.4_	33.72	52.94	13.34	3.70 ± 0.02	5.97 ± 0.02
SnS_1.2_Se_0.8_	33.94	41.38	24.68	3.73 ± 0.02	6.01 ± 0.02
SnS_0.8_Se_1.2_	33.99	27.29	38.72	3.77 ± 0.02	6.05 ± 0.02
SnS_0.4_Se_1.6_	34.16	11.07	54.77	3.80 ± 0.02	6.08 ± 0.02
SnSe_2_	33.79		66.21	3.83 ± 0.02	6.12 ± 0.02

**Table 2:** Values of O’Donnell and Chen and Bose–Einstein type fitting parameters that describe the temperature dependence of the excitonic transition energies of SnS_2-x_Se_x_ layered crystals.

Material	$E_{}^{ex}(0)$ (eV)	*S*	<*hv*> (meV)	$a_{B}$ (meV)	$\Theta_{B}$ (meV)	*dE*^ex^*/dT* (meV/K)
SnS_2_^a^	2.490	5.18	16.3	85	190	0.82
SnS_1.6_Se_0.4_^a^	2.075	4.46	13.2	78	187	0.73
SnS_1.2_Se_0.8_^a^	1.880	4.32	13.1	75	187	0.70
SnS_0.8_Se_1.2_^a^	1.679	3.48	12.3	69	185	0.61
SnS_0.4_Se_1.6_^a^	1.554	3.45	11.6	67	183	0.58
SnSe_2_^a^	1.346	3.32	10.1	64	183	0.56
SnS_2_^b^	2.559	5.76	26	151	304	
SnS_2_^c^	2.34	7.0	32.2	81	189	
SnS_1.4_Se_0.6_^c^	1.80	4.5	27.8	71	201	
SnSe_2_^c^	1.31	3.0	18.4	60	213	
SnS ^d^	1.375	1.86	24.3	41	270	

^a^ this work (piezoreflectance). ^b^ reference [38] (absorption). ^c^ reference [34] (absorption). ^d^ reference [39] (photoreflectance).

## Data Availability

The data presented in this study are available in this article.

## Sample Availability

Samples of the compounds are not available from the authors.
